# Personalized Medicine in Pediatric Urology: From Diagnosis to Individualized Risk Assessment

**DOI:** 10.3390/medsci14050570

**Published:** 2026-09-15

**Authors:** Zenon Pogorelić, Andrea Cvitković Roić

**Affiliations:** 1 Department of Pediatric Surgery, University Hospital of Split, Spinčićeva 1, 21 000 Split, Croatia; 2 Department of Surgery, School of Medicine, University of Split, Šoltanska 2a, 21 000 Split, Croatia; 3 Department of Nephrology and Urology, Helena Clinic for Pediatric Medicine, Kneza Branimira 71, 10 000 Zagreb, Croatia; 4 Faculty of Medicine, Josip Juraj Strossmayer University of Osijek, Josipa Huttlera 4, 31 000 Osijek, Croatia; 5 Faculty of Medicine, University of Rijeka, Braće Branchetta 20/1, 51 000 Rijeka, Croatia

**Keywords:** personalized medicine, precision medicine, pediatric urology, risk stratification, individualized risk assessment, congenital anomalies of the kidney and urinary tract, biomarkers, genomics, artificial intelligence

## Abstract

Background: Pediatric urology has traditionally relied on anatomical classifications, standardized diagnostic pathways, and disease-specific treatment algorithms. However, children with the same anatomical diagnosis may have substantially different risks of disease progression, renal injury, complications, and need for intervention. Personalized and precision medicine aim to integrate clinical, imaging, functional, biological, genomic, and longitudinal information to support individualized risk assessment and decision-making. Methods: This narrative review was based on a targeted literature search of PubMed/MEDLINE, Scopus, Embase and Web of Science databases. Search terms included combinations of “personalized medicine,” “precision medicine,” “risk stratification,” “pediatric urology,” “biomarkers,” “genomics,” and “artificial intelligence,” together with terms related to major pediatric urological conditions. Additional relevant publications were identified from reference lists of key articles. The literature was narratively synthesized according to its relevance to individualized risk assessment and clinical decision-making. Results: Current evidence supports an evolving role for individualized risk assessment in vesicoureteral reflux, antenatal hydronephrosis and ureteropelvic junction obstruction, congenital anomalies of the kidney and urinary tract, hypospadias, undescended testes, and disorders of sex development. Biomarkers, genomic testing, advanced imaging, and artificial intelligence may provide additional information for phenotyping and outcome prediction. However, most predictive and AI-based models remain insufficiently validated, with limitations related to external validation, calibration, reproducibility, clinical utility, and generalizability. Longitudinal reassessment is particularly important because risk may change with growth, disease progression, and treatment response. Conclusions: Personalized pediatric urology should move beyond diagnosis-based algorithms toward dynamic, risk-adapted decision-making. The goal is not to increase the number of investigations or interventions, but to identify which child is most likely to benefit from additional testing, surveillance, or treatment. Future implementation will depend on robust validation of predictive models and demonstration that personalized approaches improve clinically meaningful outcomes.

## 1. Introduction: From Diagnosis to Risk

For decades, pediatric urology has been largely organized around clearly defined diagnoses, anatomical classifications, and treatment algorithms. A child with vesicoureteral reflux (VUR) has traditionally been characterized primarily according to reflux grade, a child with antenatal hydronephrosis according to the degree of pelvic and calyceal dilatation, and a child with ureteropelvic junction obstruction (UPJO) according to a combination of anatomical and functional findings [1,2,3]. This approach has provided an important framework for standardized diagnosis and management and remains fundamental to contemporary pediatric urological practice.

However, an important consideration is that children with the same diagnosis do not necessarily have the same risk or the same clinical course, and this is why individual clinical and functional risk factors have long been incorporated into pediatric urological decision-making. Standardized classifications can describe the anatomical or functional phenotype at a particular point in time, but they do not always capture the biological heterogeneity of the individual child. In other words, diagnosis describes the present state of disease, whereas risk attempts to anticipate what may happen next [1,4].

This distinction is particularly relevant in pediatrics. The urinary tract continues to develop throughout childhood, phenotypes may change with age, and many congenital abnormalities have a highly variable natural history. Antenatal hydronephrosis, for example, is frequently transient and may resolve without intervention, whereas a smaller proportion of children have clinically significant obstruction or other pathology requiring closer surveillance or surgery [2,5]. Similarly, the clinical significance of VUR depends not only on reflux grade but also on factors such as age, recurrent febrile urinary tract infections, renal abnormalities, bladder and bowel dysfunction, and the evolution of the condition over time [3,6].

The concept of individualized risk is therefore not entirely new. Risk factors have long been incorporated into the management of VUR, hydronephrosis, and UPJO, and contemporary guidelines already recognize that management should be adapted according to clinical and functional characteristics rather than based exclusively on anatomical grade [1,2,3]. What is changing is the possibility of combining a much broader range of information, including clinical phenotype, imaging, functional measurements, biomarkers, genomic data, and longitudinal observations, to provide a more individualized estimate of clinically relevant risk [4,7]. In this review, risk does not refer to a single outcome across all pediatric urological conditions. Rather, it refers to the probability of an outcome that is clinically relevant to the condition and decision being considered, such as disease progression, renal injury or scarring, recurrent febrile urinary tract infections, need for surgical intervention, or treatment-related complications. The relevant outcome therefore differs between conditions and should always be interpreted in the context of the specific clinical question.

Importantly, a distinction should be made between established individualized decision-making and newer precision medicine approaches. In current pediatric urological practice, individualized management already relies on clinical characteristics such as age, symptoms, renal function, bladder and bowel dysfunction, anatomical findings, and changes observed during follow-up. These factors are routinely incorporated into decisions regarding surveillance and intervention in conditions such as VUR and UPJO. In contrast, biomarkers, genomic profiling, and artificial-intelligence-based prediction models should currently be regarded mainly as emerging approaches. Although these technologies may provide additional information for risk stratification and phenotyping, most remain under investigation and require further validation before they can be routinely incorporated into clinical decision-making. Their broader integration into pediatric urology should therefore be considered a future direction rather than an established component of routine care.

A considerable part of individualized decision-making is already present in everyday pediatric urological practice. Clinical factors such as age, symptoms, renal function, bladder and bowel dysfunction, anatomical findings, previous infections, and changes during follow-up are routinely used to determine whether a child should be observed, investigated further, or treated. What remains less well established is whether newer sources of information can improve this process sufficiently to change clinical decisions. Biomarkers, genomic testing, quantitative imaging, and artificial-intelligence-based prediction models have all shown potential, but the available evidence is still heterogeneous. Many studies are based on relatively small or selected cohorts, and promising diagnostic or predictive performance does not necessarily translate into clinical utility. External validation, reproducibility, calibration, and prospective evidence that these approaches improve patient management are still limited in many areas.

This creates an important gap between the growing amount of information that can be obtained from an individual child and our ability to use that information reliably in clinical practice. In addition, pediatric urological conditions often change with growth and development, meaning that risk cannot always be adequately described by a single assessment at diagnosis. A useful personalized approach therefore needs to combine established clinical assessment with emerging predictive tools and, importantly, consider how risk changes over time. The aim of this narrative review is to bring these developments together and to examine how individualized risk assessment is currently being used in pediatric urology, where emerging technologies may add value, and which approaches remain insufficiently validated for routine clinical use.

Personalized medicine therefore represents more than simply introducing new diagnostic technologies. It represents a change in how clinical decisions are framed. Instead of asking only, What disease does this child have?, the more relevant questions become What is the risk for this particular child, how is that risk likely to evolve, and can our intervention meaningfully change the outcome? This concept is already reflected in routine pediatric urological practice through the integration of clinical, functional, anatomical, and longitudinal characteristics into management decisions. Newer approaches, including biomarkers, genomic testing, artificial intelligence, and individualized prediction models, may further refine this process, but their clinical implementation remains at different stages of development and validation [4,7,8,9].

Importantly, personalized medicine should not be interpreted as a justification for performing more investigations or interventions. The objective is quite the opposite: to identify which children are most likely to benefit from additional investigation or treatment while avoiding unnecessary procedures in those at low risk. As these approaches develop, the challenge for pediatric urologists will increasingly be to integrate these new sources of information without losing the central role of clinical judgment and longitudinal observation [4,7].

This shift from diagnosis toward individualized risk assessment may become one of the defining developments in pediatric urology. The future is unlikely to be about doing more to every child with a particular diagnosis, but about identifying which child actually needs more and which child can safely have less.

## 2. Methods

This narrative review was based on a targeted literature search of PubMed/MEDLINE, Scopus, Embase and Web of Science databases. The search was performed in July 2026 using combinations of keywords related to “personalized medicine”, “precision medicine”, “risk stratification”, “pediatric urology”, “vesicoureteral reflux”, “hydronephrosis”, “ureteropelvic junction obstruction”, “CAKUT”, “hypospadias”, “genomics”, “biomarkers”, and “artificial intelligence”. Additional relevant publications were identified by screening the reference lists of key articles and recent reviews.

The literature was selected based on relevance to the clinical application of individualized risk assessment, precision medicine, and emerging diagnostic or predictive approaches in pediatric urology. Priority was given to clinical studies, systematic reviews, clinical guidelines, and studies evaluating prediction models, biomarkers, genomic testing, and artificial intelligence, with particular attention to publications addressing current developments in these fields. Additional studies were identified through screening the reference lists of key articles and reviews and were included when considered directly relevant to the scope of the review.

## 3. What Does Personalized Medicine Mean in Pediatric Urology?

The terms personalized medicine, precision medicine, and risk-based decision-making are related but emphasize different aspects of clinical decision-making. Precision medicine seeks to improve the accuracy of medical decisions by tailoring prevention, diagnosis, treatment, and prognosis to biological and clinical characteristics shared by relevant patient subgroups. Personalized medicine places greater emphasis on tailoring clinical recommendations to the individual patient’s biological and clinical characteristics, while also considering relevant patient and family circumstances [10]. Risk-based decision-making, in contrast, places estimates of the likelihood and severity of clinically relevant outcomes at the center of decision-making, with explicit consideration of uncertainty [11]. In this review, individualized risk assessment is considered the practical bridge through which clinical decision-making.

In pediatric urology, these concepts come together in a straightforward clinical principle: the same anatomical diagnosis does not necessarily carry the same clinical significance, or require the same management, in every child. In VUR, for example, decisions are influenced not only by the grade of reflux, but also by the child’s age and sex, history of febrile urinary tract infections, renal morphology and function, laterality, bladder and bowel dysfunction, and the subsequent clinical course. This has increasingly shifted VUR management away from a purely grade-based approach toward individualized risk assessment, in which surveillance, antibiotic prophylaxis, and surgical intervention are considered in the context of the child’s overall risk profile [3,6,12,13,14].

The same principle applies to UPJO. Children with a similar degree of hydronephrosis may follow very different clinical courses. Symptoms, urinary drainage, differential renal function, renal parenchymal appearance, and changes observed over time can all influence the significance of the obstruction and the decision to intervene. Thus, the extent of hydronephrosis alone provides only a partial picture of the individual child’s risk and should be interpreted together with functional and longitudinal findings [1,15,16].

**Figure 1:**
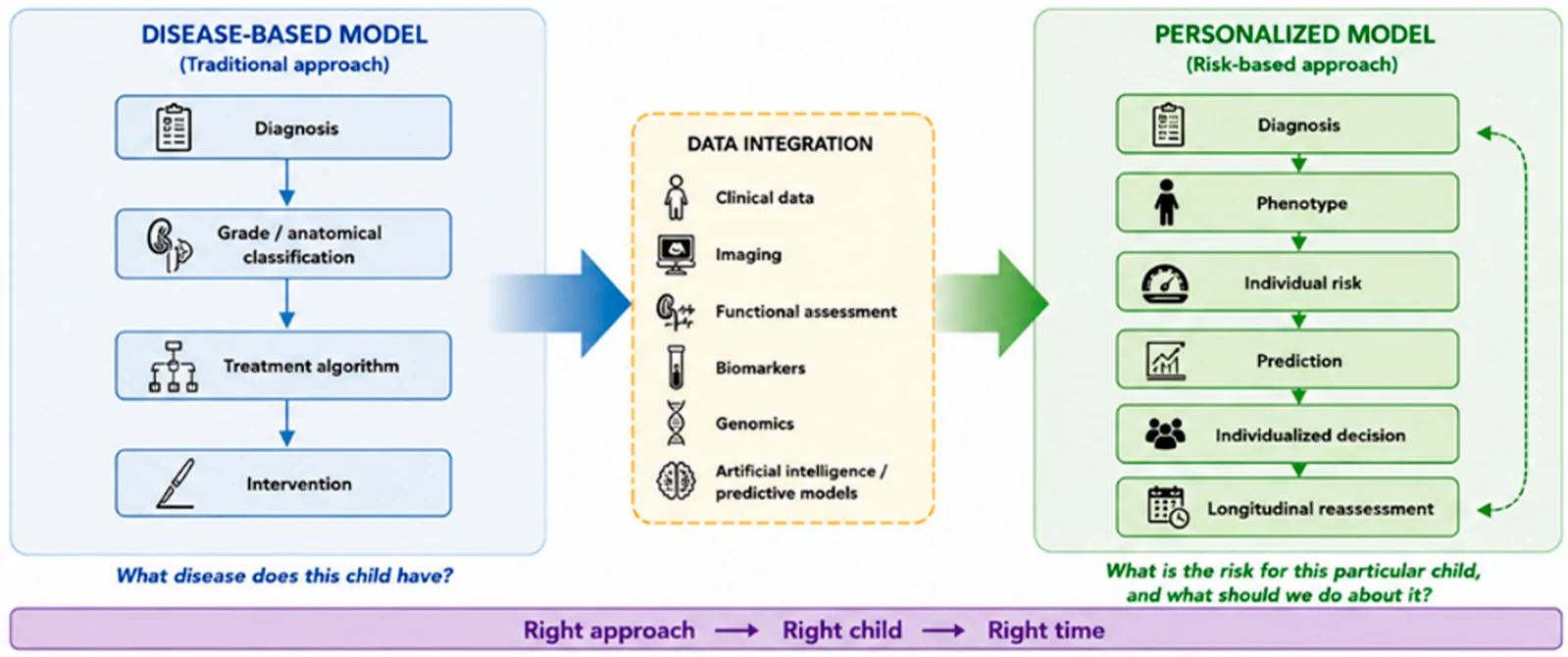
From disease-based algorithms to personalized pediatric urology.

Personalized medicine seeks to integrate these dimensions into a clinically meaningful estimate of risk. It also recognizes that the optimal decision is not necessarily the most aggressive one. In a child with a very low probability of progression, avoiding unnecessary imaging, antibiotics, anesthesia, or surgery may represent a highly personalized treatment strategy. In this sense, personalized medicine is not medicine with more tests. It is medicine with better targeted tests and better decisions (Figure 1).

The main domains through which this approach may evolve in pediatric urology are summarized in Table 1, highlighting the transition from isolated measurements and protocol-driven management toward integrated, longitudinal, and risk-adapted decision-making.

## 4. From Disease-Based Algorithms to Individual Risk Profiles

VUR provides one of the clearest examples of the transition from an anatomically based disease classification toward risk stratification based on individual patient characteristics. Historically, reflux grade has played a central role in management decisions. However, grade alone does not determine whether a child will experience recurrent febrile urinary tract infections, develop renal scarring, or ultimately require surgery. The clinical context and the presence of renal abnormalities substantially modify the individual risk profile [3,6,12,13,14].

This becomes particularly important when the ultimate objective is preservation of renal function rather than simply correction of an anatomical abnormality. The clinical challenge is to distinguish children in whom reflux represents a meaningful risk from those in whom the probability of clinically important renal injury is low.

At the same time, the increasing availability and technical simplicity of endoscopic treatment have introduced a different challenge: the possibility of treating children who may not require treatment at all. The widespread adoption of endoscopic injection, particularly with dextranomer/hyaluronic acid, has made intervention relatively straightforward, minimally invasive, and attractive to both clinicians and families [17]. However, the fact that a procedure is technically simple and associated with low short-term morbidity does not necessarily mean that it is indicated. A substantial proportion of children with primary VUR, particularly those at lower risk, will experience spontaneous resolution or remain clinically stable without surgical correction. Consequently, the availability of an easy endoscopic option may potentially lower the threshold for intervention and thereby contribute to treatment of some children whose long-term risk is limited [18,19,20].

This is an important example of why personalized medicine should not simply provide more options for intervention, but should help determine which children should not be treated. The relevant question is therefore not whether reflux can be corrected endoscopically, but whether correcting reflux in a particular child is expected to provide a meaningful clinical benefit that outweighs the burden and potential risks of intervention.

The same principle applies in the opposite direction. Endoscopic treatment is often perceived as the natural first choice whenever intervention is considered because it is less invasive and can usually be performed as a short procedure. However, the least invasive procedure is not necessarily the most appropriate definitive treatment for every child. Endoscopic injection has variable success according to reflux grade, anatomy, laterality, bladder and bowel dysfunction, and other patient-related factors. In children with high-grade reflux, complex anatomy, recurrent febrile urinary tract infections despite appropriate management, or an indication for definitive correction, open ureteral reimplantation may offer higher definitive success in selected children than repeated endoscopic procedures [19,20,21]. Choosing endoscopy simply because it is less invasive may therefore represent another form of non-personalized, procedure-driven decision-making.

The personalized approach should consequently move beyond the question of which procedure is less invasive toward the more clinically relevant question of which treatment is most appropriate for this individual child. In some children, the correct intervention may be no intervention at all; in others, endoscopic treatment may represent an appropriate balance between efficacy and invasiveness; while in selected children, proceeding directly to definitive ureteral reimplantation may avoid repeated procedures and provide a more reliable long-term solution. The objective should be to match the intensity and type of treatment to the child’s individual risk rather than to the availability or technical attractiveness of a particular procedure.

The search for biomarkers capable of identifying renal injury illustrates the next step in this evolution. A variety of urinary and serum markers have been investigated, including markers of tubular injury, inflammation, and glomerular dysfunction. Recent systematic reviews have evaluated biomarkers such as NGAL, cystatin C, interleukin-6, and other candidates for the detection or prediction of renal scarring in children with VUR [22,23]. Earlier studies also investigated kidney injury molecule-1 as a potential noninvasive marker of renal scarring [24].

More importantly, recent work has moved beyond individual biomarkers toward combined biomarker models. Models incorporating several renal biomarkers have shown potential for improving prediction of renal scarring compared with individual markers alone [25]. This is conceptually important because biological processes such as renal scarring are unlikely to be represented adequately by a single molecular signal.

Nevertheless, the current evidence remains insufficient for routine clinical implementation. Differences in patient selection, timing of sample collection, assay methodology, definitions of renal scarring, and external validation remain important limitations. Biomarkers therefore currently represent promising components of personalized risk assessment rather than replacements for established clinical and imaging pathways.

The broader lesson is clear: a biomarker is valuable not because it is biologically interesting, but because it improves a clinical decision.

## 5. Hydronephrosis and UPJO: Predicting What Happens Next

A similar shift is taking place in the management of antenatal hydronephrosis and UPJO. The initial diagnosis of hydronephrosis is usually straightforward with modern ultrasonography. The more difficult question comes afterward: which children are likely to remain stable, which are likely to deteriorate, and which will eventually benefit from surgery? Current management therefore increasingly focuses on the combination of ultrasound findings, renal function, and the clinical course rather than on a single imaging finding [1,26,27].

This distinction is particularly important in infants and young children, in whom hydronephrosis or suspected UPJO does not, by itself, constitute an indication for immediate surgery. Many children can be followed safely when they remain asymptomatic, maintain preserved differential renal function, and show stable or improving hydronephrosis over time [1,26,27]. The aim is not simply to make the ultrasound look normal as quickly as possible, but to preserve renal function and recognize the child whose course is becoming unfavorable. In this setting, the evolution of hydronephrosis and renal function over time may be more informative than the degree of dilatation recorded at a single examination.

Conversely, intervention becomes more compelling when there is deterioration in renal function, progressive parenchymal thinning, worsening drainage, recurrent symptoms, or other evidence that the affected kidney is being compromised [1,15,26,27]. The clinical question is therefore not simply whether UPJO is present, but whether the obstruction is clinically significant and whether intervention at that particular stage is likely to provide a meaningful benefit. A recent systematic review also found that preoperative differential renal function is among the more consistent predictors of renal functional recovery after pyeloplasty, although the available evidence remains heterogeneous [27].

Even when decompression is required, definitive reconstruction does not necessarily have to be performed immediately in every high-risk infant. Temporary urinary drainage may have a role in selected children with severe obstruction and very poor renal function, particularly when the potential for functional recovery remains uncertain. In such situations, a temporary JJ stent or percutaneous nephrostomy can provide decompression while allowing renal function and the clinical course to be reassessed before definitive reconstruction [15,28]. This should be regarded as a selective strategy rather than a routine alternative to pyeloplasty. The decision to decompress temporarily, proceed directly to pyeloplasty, or continue observation should depend on the clinical context and the expected benefit of each approach.

Current assessment therefore brings together ultrasound findings, symptoms, drainage patterns, differential renal function, and changes observed over time. No single parameter provides a perfect prediction of future renal deterioration or the eventual need for surgery [1,26,27]. This uncertainty is one reason why some children undergo repeated imaging and functional testing despite ultimately following a benign course, whereas others show progressive deterioration before the need for intervention becomes clear.

Urinary biomarkers have been investigated as a possible way of adding biological information to this clinical assessment. Studies have examined markers including NGAL, KIM-1, MMP-7, TIMP-2, and CA19-9 [29,30,31,32]. Although several markers have shown associations with obstruction or impaired renal function, the findings have not been sufficiently consistent to support their routine use in clinical practice. Differences in study populations, assay methods, diagnostic thresholds, and definitions of obstruction remain important limitations [29,30,31,32].

One particularly attractive approach is to combine biomarkers with imaging rather than considering them as competing diagnostic strategies. Ultrasound provides the anatomical picture, functional assessment provides information about drainage and renal function, while biomarkers may add information about the biological response to obstruction. Studies combining urinary biomarkers with ultrasound parameters have suggested that biomarkers may provide additional discriminatory information beyond ultrasound alone [29,30]. More recent prospective data on urinary CA19-9/creatinine further support the potential value of biomarkers as an adjunct to conventional assessment, although their clinical role remains to be established. Importantly, the degree of clinical readiness differs among the investigated biomarkers. For example, a recent prospective study of urinary CA19-9/creatinine found higher levels in children with hydronephrosis, but the marker did not correlate with hydronephrosis severity or dynamic renal scintigraphy findings and did not reliably predict the need for surgery [32]. Thus, although CA19-9/creatinine is a promising non-invasive biomarker, its current evidence supports potential clinical relevance rather than routine clinical use.

These findings support further investigation of multimodal prediction models that combine biomarkers with imaging and functional data rather than relying on a single marker.

Time adds another important dimension. A single ultrasound examination provides a snapshot, whereas repeated examinations provide a trajectory. This longitudinal perspective is increasingly being explored using machine-learning approaches. The Hydronephrosis Severity Index, developed using deep learning applied to pediatric ultrasound images, showed strong predictive performance across multiple pediatric institutions, including external institutional testing [33]. More recently, Khondker et al. used deep learning to predict the likelihood of surgical intervention in infants with hydronephrosis from ultrasound images [34]. Interestingly, incorporating multiple ultrasound examinations did not necessarily improve prediction compared with a single ultrasound examination. In other words, more longitudinal information is not automatically better information; its value depends on whether it actually improves clinical prediction.

Ultimately, the management of antenatal hydronephrosis and UPJO may increasingly move from an anatomy-driven model toward a risk- and trajectory-based model. The key question is not simply how abnormal the collecting system looks today, but whether the individual child is demonstrating evidence of clinically meaningful obstruction and whether intervention is likely to prevent future renal damage. In some children, this will mean earlier pyeloplasty; in others, prolonged observation with preserved renal function may be the most appropriate treatment. For selected high-risk infants, temporary decompression may provide a bridge to definitive treatment. This is where personalized medicine can add real clinical value: not by generating more measurements, but by helping us decide which measurements matter, when intervention is justified, and which children can safely wait.

## 6. Genomics: When a Urological Diagnosis Is Part of a Larger Disorder

Genomic medicine represents another important component of personalized pediatric urology, particularly in children with congenital anomalies of the kidney and urinary tract (CAKUT).

CAKUT encompasses a highly heterogeneous group of disorders, ranging from renal agenesis and hypodysplasia to abnormalities of the collecting system [35]. Genetic studies have identified numerous genes and molecular pathways involved in kidney and urinary tract development [36]. At the same time, the genetic contribution varies substantially between phenotypes, and a genetic diagnosis is not expected in every child with an isolated structural abnormality [37].

Genomic testing becomes particularly relevant when the clinical picture is more complex—for example, in children with severe or bilateral disease, early kidney dysfunction, extrarenal abnormalities, a positive family history, or an otherwise atypical phenotype. In a cohort of children with CAKUT diagnosed within the first 1000 days of life, whole-exome sequencing identified a pathogenic or likely pathogenic variant in approximately one-quarter of patients, illustrating the potential diagnostic value of genomic testing in appropriately selected patients [38]. More recent data from children with non-isolated CAKUT similarly suggest that clinical exome sequencing can provide a meaningful diagnostic yield, particularly when renal abnormalities occur together with developmental, neurological, or other extrarenal features [39,40,41].

The value of genomic testing, however, may extend beyond simply naming the underlying disorder. A genetic diagnosis can draw attention to previously unrecognized extrarenal manifestations and may influence subsequent surveillance, management, and evaluation of family members [41]. In this respect, genomic information can change the clinical picture rather than merely explain it.

The concept of reverse phenotyping is particularly relevant. Once a potentially pathogenic variant has been identified, the clinician can return to the patient and actively look for clinical features associated with the corresponding syndrome. In a large CAKUT cohort, this approach helped identify previously unrecognized syndromic features in a proportion of children with a molecular diagnosis [39]. The process is therefore bidirectional: the phenotype can guide genomic testing, while the genomic finding can refine the phenotype.

Recent work has further strengthened the case for clinical exome sequencing in children with complex or non-isolated CAKUT. In a cohort of more than 500 individuals with CAKUT+, clinical exome sequencing established a molecular diagnosis in 27.4% of cases, and many of the diagnoses would not have been captured by currently available CAKUT-focused gene panels [40]. This is particularly relevant in pediatric urology, where the initial presentation may be dominated by an apparently isolated renal or urinary tract abnormality.

For the pediatric urologist, this changes the way a congenital renal anomaly can be viewed. A child with renal hypodysplasia, a solitary kidney, urinary tract malformation, or another CAKUT phenotype may not simply have an anatomical problem requiring anatomical management. In some cases, the urinary phenotype may be one component of a broader genetic disorder. Recent reviews of genomic testing in pediatric urology emphasize that recognizing this possibility can affect diagnosis, surveillance, family evaluation, and, in selected disorders, treatment [41].

At the same time, genomic testing should not become indiscriminate. Variant interpretation can be challenging, and results may include variants of uncertain significance or unexpected findings that have implications for the child and family. Genetic counseling and appropriate pre- and post-test discussion are therefore important parts of the process. More broadly, translating precision health into pediatric practice raises questions about evidence generation, cost-effectiveness, equity, and implementation that extend beyond the technical ability to sequence a patient’s genome [42]. The current clinical utility of genomic testing appears to be greatest in children with complex, bilateral, syndromic, familial, or otherwise atypical CAKUT phenotypes, where identifying an underlying genetic diagnosis may influence diagnostic evaluation, surveillance strategies, and family counseling. In contrast, broad genomic testing in children with isolated, non-syndromic anomalies is less likely to provide immediate clinical benefit and should be considered within an appropriate clinical context.

Genomic medicine may therefore move pediatric urology from phenotype recognition toward etiological precision, but only when genomic information can meaningfully change how the child is understood, monitored, or treated.

## 7. External Genitalia: Beyond Anatomical Classification

Hypospadias is another area in which pediatric urology is gradually moving away from purely anatomical classification toward a more individualized approach. Traditional descriptions have focused primarily on meatal position, degree of chordee, glans configuration, and penile size. These features remain essential for surgical planning, but they do not fully capture the complexity of the condition or predict long-term functional and patient-reported outcomes. Children with apparently similar anatomical forms of hypospadias may have substantially different surgical challenges, complication risks, and functional consequences [1,43].

The concept of personalized medicine is particularly relevant because the goal of hypospadias surgery is not simply to relocate the meatus. The ultimate objectives are a functional urinary stream, adequate penile straightening, preservation of sexual function, acceptable genital appearance, and satisfactory psychosocial outcomes. These outcomes may not always correlate directly with the initial anatomical classification. The choice of reconstructive strategy may be influenced by anatomical and patient-specific factors, including penile size, tissue quality, degree of curvature, associated anomalies, previous surgery, and age at reconstruction, while technical factors such as suture material may also affect postoperative outcomes [43,44]. The need for individualized decision-making becomes particularly clear in redo surgery, where previous repair may substantially alter the anatomy and available tissue. Recent evidence comparing surgical approaches for distal urethral strictures after failed hypospadias repair further supports tailoring reconstruction to the individual clinical situation rather than relying on a single preferred technique [45].

Importantly, this represents an example of personalized care that extends beyond the use of diagnostic or predictive technologies. In hypospadias, personalization also means defining treatment success according to outcomes that are meaningful for the individual child, rather than relying solely on anatomical correction or surgeon-reported outcomes.

This raises an important question for future pediatric urology: should surgical selection be based primarily on anatomical classification or also on the predicted risk of complications and the functional goals of the individual child? Future decision-making may increasingly incorporate this broader patient-specific phenotype rather than relying on fixed anatomical categories alone [43,46,47]. Anatomy therefore remains important, but may not be sufficient as the sole determinant of surgical choice.

This also means avoiding a rigid adherence to established surgical “recipes” or assuming that the most widely adopted technique is necessarily the best option for every child. For example, in selected children with distal hypospadias, tubularized incised plate (Snodgrass) urethroplasty should not necessarily be considered the default approach in every child with distal hypospadias when alternative techniques may provide comparable outcomes in appropriately selected anatomy [47,48]. The principle is not to promote one technique over another, but to select the simplest reliable procedure that is appropriate for the individual anatomy and goals of that child. In this context, the most sophisticated approach is not necessarily the most extensive reconstruction, but the procedure that provides the best balance between functional outcome, complication risk, tissue preservation, and the child’s long-term quality of life.

A similar principle applies to undescended testes. Although the diagnosis is anatomical, the long-term consequences are biological and functional, including impaired fertility potential, altered testicular development, and increased malignancy risk. Timing of orchidopexy has therefore increasingly been considered in relation to the developmental trajectory of the testis rather than simply the presence of an undescended testis [49]. The individualized assessment should consider factors such as testicular position and palpability, size, unilateral or bilateral involvement, associated anomalies, and any suspicion of a disorder of sex development, as these may influence the diagnostic and surgical approach. However, such individualization should not lead to unnecessary delay of orchidopexy beyond the timeframe recommended by current guidelines [1,49,50,51].

The same concept becomes even more evident in disorders of sex development (DSD) and complex genital anomalies. Here, personalized medicine is inherently multidisciplinary. Anatomical findings need to be interpreted together with endocrine, genetic, hormonal, reproductive, and psychosocial information. Genomic testing may identify the underlying disorder, while endocrine evaluation can characterize the functional phenotype and guide management. Surgical intervention, when indicated, should therefore be considered within a broader individualized care pathway rather than as an isolated anatomical correction [52,53,54].

These conditions also illustrate an important difference between pediatric urology and some other surgical specialties: the success of treatment cannot always be measured by an anatomical endpoint. A technically successful reconstruction may not necessarily translate into the best long-term functional or patient-reported outcome. Future personalized pediatric urology will therefore need to incorporate outcomes that matter to the child, including urinary function, sexual function, body image, psychosocial well-being, and quality of life [43,55]. This shift toward patient-reported outcomes is particularly important because some aspects of success are difficult to capture with a conventional surgical examination alone.

The field of external genital reconstruction thus provides another important example of the transition from “What does the anatomy look like?” to “What outcome matters for this child, and which intervention is most likely to achieve it?”.

Examples of personalized decision-making across pediatric urology are summarized in Table 2.

## 8. Beyond Single Biomarkers: Building a Multimodal Phenotype

The future of personalized medicine is unlikely to depend on the discovery of a single biomarker that solves a particular clinical problem. A more realistic model is the development of a multimodal individual risk profile, in which information from different clinical domains is combined to provide a more complete picture of the individual patient [35,36,56,57].

Such a profile could integrate clinical characteristics, age and developmental stage, imaging findings, functional measurements, laboratory and urinary biomarkers, genetic information, previous urinary tract infections, response to treatment, and longitudinal changes over time. In the future, information derived from digital health technologies may further expand this profile. Recent advances in artificial intelligence have already shown that clinical variables and imaging features can be used together to improve risk stratification in pediatric hydronephrosis and other urological conditions [9,57,58,59,60].

The purpose of such integration is not simply to accumulate more information. It is to transform multiple imperfect measurements into a clinically meaningful prediction. This distinction is important: adding variables does not automatically make a prediction better. The value lies in identifying which combination of signals provides information that is relevant to the clinically relevant decision. Recent work in pediatric urology illustrates both the potential and the limitations of this approach, with machine-learning models showing promising discrimination but still requiring external validation and assessment of reproducibility before routine clinical use [9,57,59].

Consider two children with apparently similar hydronephrosis on ultrasound. One may have stable renal function, improving dilatation, reassuring longitudinal measurements, and no clinical symptoms. The other may demonstrate progressive dilatation, deteriorating functional parameters, and evidence of renal injury. Anatomically, they may appear similar at a single point in time. Their individual risks, however, may be fundamentally different. A multimodal approach may therefore help move the assessment from describing how abnormal the kidney looks to estimating what is likely to happen next. This principle is already being explored in CAKUT, where multicenter machine-learning models have incorporated clinical and congenital-hereditary characteristics to estimate the risk of future kidney failure at multiple time points [59].

Biomarkers may also contribute to this broader risk profile, particularly when they are interpreted alongside established clinical and imaging parameters rather than in isolation. Systematic reviews of biomarkers for renal scarring in children with vesicoureteral reflux have highlighted both the potential diagnostic value of urinary and serum markers and the substantial heterogeneity between individual studies [22,23]. This supports the broader concept that biomarkers are more likely to become clinically useful as components of integrated prediction models than as stand-alone tests. This distinction is central to personalized pediatric urology.

The future therefore lies less in searching for a single “perfect” test and more in understanding how multiple relatively modest signals can be combined to produce a clinically useful estimate of risk. Importantly, this does not necessarily mean that every child should undergo more tests. In some situations, the most useful personalized approach may be to identify a small number of high-value variables and use them consistently, rather than continuously expanding the diagnostic work-up. The recent development of AI-based models in pediatric urology, including approaches using ultrasound-derived information and other clinical data, suggests that this shift from isolated measurements toward integrated prediction is already beginning [9,57,58,61].

## 9. Artificial Intelligence: From Image Analysis to Clinical Prediction

Artificial intelligence is particularly relevant to personalized medicine because it can integrate multiple clinical, imaging, and biological variables and identify patterns that may be difficult to capture with conventional statistical methods. In pediatric urology, its use has expanded rapidly, with applications ranging from image interpretation and disease classification to prediction of surgical outcomes and postoperative complications [62]. The development of the AI-PEDURO collaborative has further contributed to this field by providing a living evidence synthesis and repository of artificial intelligence and machine-learning models in pediatric urology [9]. Recent updates of the AI-PEDURO collaborative demonstrate a rapidly expanding number of AI and machine-learning models in pediatric urology, particularly in hydronephrosis, pyeloplasty, vesicoureteral reflux, and urinary tract infection [9,57].

This distinction between technical performance and clinical usefulness is essential. A model that performs well in its development cohort does not necessarily retain the same accuracy in a different clinical setting. Differences in patient characteristics, disease prevalence, imaging equipment, acquisition protocols, measurement practices, and clinical decision-making can all influence model performance. Consequently, external validation in independent and clinically relevant populations should be regarded as an essential step before AI models are incorporated into routine pediatric urological practice [9,57]. Assessment should therefore extend beyond discrimination to include calibration, external validation, reproducibility, clinical utility, and the potential impact of model use on patient outcomes.

A similar distinction applies to artificial intelligence. The Hydronephrosis Severity Index, developed using deep learning applied to pediatric ultrasound images, showed strong predictive performance across multiple pediatric institutions, including external institutional testing [33]. Similarly, a multicenter deep-learning study evaluated prediction of surgical intervention using ultrasound data from multiple clinical visits [34]. These findings represent an important step toward clinical application; however, predictive performance alone does not establish clinical utility. Further prospective evaluation is needed to determine whether implementation of such models improves clinical decision-making, reduces unnecessary follow-up or imaging, or improves patient outcomes before routine clinical adoption.

Recent studies illustrate both the potential and the current limitations of these approaches [57,58,59,60,61,62,63,64]. Deep-learning methods have been developed to classify the severity of hydronephrosis from ultrasound images and to predict the need for surgical intervention using longitudinal imaging data [34,63]. Other machine-learning models have explored prediction of postoperative urinary tract infection after pediatric pyeloplasty, including explainable approaches designed to identify the clinical variables contributing most strongly to individual predictions [64]. More experimental work has combined deep learning with computational fluid dynamics to derive non-invasive measures of urinary flow dynamics in children with hydronephrosis, illustrating how AI may eventually extend beyond image classification toward the characterization of underlying pathophysiological processes [65].

An emerging area of interest is the use of AI-assisted image analysis together with three-dimensional reconstruction in pediatric renal tumors. Patient-specific 3D models can provide a more detailed representation of tumor volume and its relationship to the renal parenchyma, vessels, and collecting system, potentially improving preoperative planning for nephron-sparing surgery. AI-based segmentation has also been investigated as a means of automating the reconstruction of Wilms tumors and the affected kidney, although current approaches still require further validation before routine clinical use [66]. In this context, the potential value of AI lies not simply in producing more sophisticated images, but in transforming imaging data into patient-specific anatomical information that can help surgeons assess the feasibility and extent of renal preservation. In the longer term, integration of imaging features with clinical, pathological, and potentially genomic data may allow more individualized prediction of tumor behavior and treatment response.

These developments are promising, but technological sophistication should not be equated with clinical readiness. The important question is not whether an AI model can achieve high discrimination in a retrospective dataset, but whether its use changes clinical decision-making in a way that benefits patients. The real clinical value of AI will therefore depend on prospective and external validation, transparent reporting of errors and uncertainty, and demonstration that its use can improve outcomes, reduce unnecessary investigations, or improve the timing and selection of interventions [10,57].

The ultimate goal should not be to replace the pediatric urologist. Rather, AI should provide the pediatric urologist with better, more individualized information at the moment when a clinical decision has to be made.

## 10. Personalized Surgery: The Question Is Not Only “What?” but “Who?” and “When?”

Surgery is perhaps the area in which personalized medicine can have the most immediate practical impact. Advances in endoscopic, laparoscopic, robotic, and other minimally invasive techniques have substantially expanded the range of procedures available to pediatric urologists [67,68,69]. However, technical progress does not by itself answer the more fundamental question: which child actually needs an intervention?

Traditional surgical decision-making has often focused on defining the most appropriate procedure for a given anatomical abnormality. A personalized approach shifts the emphasis toward the individual risk of clinically meaningful disease progression and the expected benefit of intervention. In a child at low risk of progression, continued observation may be the most appropriate treatment, whereas in a child with a high probability of progressive renal damage, delaying intervention may carry greater risk [11,26,27]. The decision is therefore not simply which procedure to perform, but whether intervention is necessary, when it should be undertaken, and which treatment is most likely to provide meaningful benefit with the lowest burden.

This principle is particularly relevant to UPJO and VUR, where anatomical severity alone does not always determine the need for surgery. The objective should not be to correct every radiological or anatomical abnormality, but to identify children in whom intervention is likely to prevent clinically important harm [18,19,20,21,26,27,28].

In this context, minimally invasive surgery should not become an end in itself. The availability of a more advanced surgical technique does not necessarily mean that it is the best choice for every patient. The most appropriate operation is therefore the one that provides the greatest expected benefit for the individual patient while minimizing procedural burden and long-term harm [69,70].

## 11. Dynamic Risk and Longitudinal Reassessment

Perhaps one of the most important conceptual contributions of personalized medicine is the recognition that the pediatric patient is not a static phenotype.

Children grow, develop, and change over time. Hydronephrosis may resolve, remain stable, or progress. VUR may disappear spontaneously, while in other children it may become clinically relevant because of recurrent febrile urinary tract infections. Bladder function may improve with maturation or, conversely, become an important contributor to recurrent infections and renal risk. Renal function may remain stable for years, but it may also deteriorate in a subset of children [2,5,20,71].

Risk should therefore also be regarded as dynamic rather than fixed. A child initially classified as low risk may move into a higher-risk category because of recurrent febrile infections, increasing hydronephrosis, deterioration in renal function, or newly recognized lower urinary tract dysfunction. Conversely, a child initially considered to be at higher risk may demonstrate stability or spontaneous improvement and may subsequently require less intensive surveillance [20,71,72].

This concept supports a future model of adaptive surveillance, in which the intensity and timing of follow-up are adjusted according to the child’s evolving clinical course rather than determined exclusively by the initial diagnosis or risk category. Sequential ultrasound and functional assessment already reflect elements of this approach in children with hydronephrosis and suspected obstruction, where changes over time may be more informative than a single measurement [5,26,72].

Such an approach could reduce unnecessary investigations in children who remain clinically stable while allowing earlier reassessment and intervention in those whose trajectory becomes concerning. The aim is not simply to perform fewer tests, but to perform the right test at the right time for the right child.

The fundamental shift is therefore from asking “When is the next scheduled test?” to asking “Has this child’s risk changed enough to justify another test?”

## 12. Challenges and Ethical Considerations

Despite its promise, personalized medicine should not be adopted uncritically. The first challenge is data quality. Many pediatric urological conditions are relatively uncommon, making sufficiently large and representative datasets difficult to assemble. When small datasets are combined with a large number of predictor variables, the risk of overfitting becomes substantial, and apparently impressive model performance may not translate into reliable predictions in new patients [9,10,57,73].

The second challenge is external validation. Predictive models need to demonstrate that they perform reliably across institutions, populations, imaging platforms, and clinical environments. This is particularly important for AI-based models, for which good technical performance does not necessarily translate into clinical usefulness or improved patient outcomes [9,57,58,73]. For AI and multi-marker models, demonstration of discrimination in a development cohort should therefore be regarded as an initial step rather than evidence of clinical readiness; independent external validation, assessment of calibration and reproducibility, and prospective evaluation of clinical utility are needed before these models can reliably support clinical decisions.

The third challenge is interpretability. Clinicians need to understand not only what a model predicts, but also why the prediction was made and how reliable it is in an individual patient. A prediction of a high probability of disease progression is meaningful only if the clinician understands the uncertainty surrounding that estimate and how it should influence management. Transparent reporting and appropriate evaluation of prediction models are therefore essential if these tools are to support rather than complicate clinical decision-making [10,57,73].

The fourth challenge is equity. AI systems trained predominantly on selected populations may perform less well in underrepresented groups and may inadvertently reproduce existing healthcare disparities. Similarly, access to genomic testing and other advanced diagnostic technologies can vary considerably between healthcare systems and patient populations [42,56,74]. Economic and practical considerations are also important for implementation. Genomic testing, advanced imaging, and AI-based platforms may require substantial laboratory infrastructure, computational resources, specialized personnel, and ongoing technical support. These requirements may limit their availability outside well-resourced centers and can make widespread implementation difficult in healthcare systems with constrained budgets. In such settings, precision approaches will need to be adapted to available resources, with priority given to tools that provide clear clinical benefit and can be integrated into existing workflows. Otherwise, the introduction of increasingly sophisticated technologies could unintentionally widen differences in access to pediatric urological care.

At the point of care, multimodal information should therefore be filtered according to its expected clinical value rather than incorporated automatically into every decision. A practical approach is to first define the clinical question and the decision that needs to be made, then consider whether each additional data source is likely to change risk classification or management. Findings that are unlikely to alter the clinical decision should not automatically trigger further testing or follow-up. This stepwise approach may help clinicians distinguish actionable signals from background information and reduce the risk of false-positive cascades, incidental findings, and unnecessary interventions. Importantly, decision-support tools should present a limited number of clinically relevant outputs rather than requiring clinicians to interpret multiple independent measurements, thereby reducing cognitive and administrative burden and facilitating integration into routine workflows.

Genomic medicine introduces additional ethical considerations, including incidental findings, variants of uncertain significance, genetic counseling, data ownership, privacy, and the potential implications of genetic findings for family members [38,39,40,41,42,75].

More data do not necessarily lead to better decisions. Increasingly sophisticated imaging, biomarkers, genomic information, and AI-based predictions may increase testing, cost, burden, incidental findings, and false-positive results without improving outcomes. The key question should therefore be which information will meaningfully change management. Personalized care should remain proportionate to the child’s risk and expected clinical benefit [4,10,42,74]. A further challenge is implementation: even well-validated models may face barriers related to integration into clinical workflows, interoperability with existing health information systems, cost, regulatory requirements, and clinician acceptance.

## 13. The Future of Decision-Making in Pediatric Urology

The future of pediatric urology is unlikely to be defined by a single technology. Instead, it will probably involve a gradual shift from disease-centered algorithms toward more individualized and dynamic decision-making [7,10,76].

The traditional pathway can be represented as Diagnosis → Grade → Algorithm → Intervention. The emerging model is more appropriately represented as Diagnosis → Phenotype → Risk → Prediction → Individualized decision → Longitudinal reassessment.

Genomics may help explain why a child has a particular condition. Advanced imaging and biomarkers may provide a better understanding of what is happening, while artificial intelligence may help estimate what is likely to happen next. The pediatric urologist, however, will remain responsible for deciding what should be done about it [8,56,77].

This distinction is important. Personalized medicine will not eliminate clinical judgment; rather, its purpose is to make clinical judgment better informed. The transition toward personalized medicine should therefore not be viewed as a technological revolution occurring outside everyday pediatric urology. It is better understood as an evolution of the decision-making process itself [76,78].

Ultrasound, assessment of renal function, clinical history, and careful longitudinal observation will remain fundamental components of pediatric urological care. Genomics, biomarkers, advanced imaging, and AI will increasingly provide additional layers of information when they can meaningfully refine individual risk or alter management [8,22,23,24,25,33,34,57,58,59,60].

**Figure 2:**
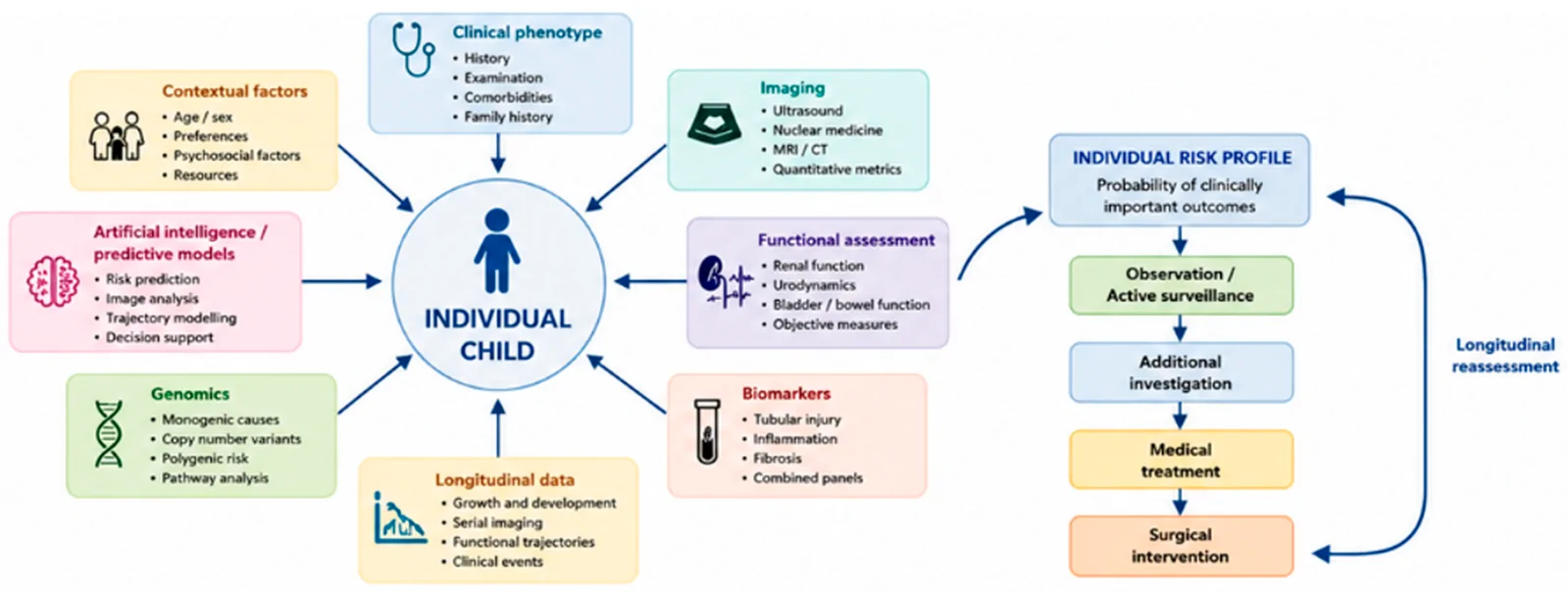
The building blocks of individualized risk assessment in pediatric urology.

The most sophisticated future system may therefore be one that sometimes recommends more investigation and, in selected patients, earlier intervention, but also recognizes when less intervention is the better decision because the predicted risk is low. In that sense, the success of personalized pediatric urology should ultimately be measured not by how much technology is used, but by how effectively available information is translated into the right decision for the individual child [76,77]. The building blocks of individualized risk assessment in pediatric urology is presented in Figure 2.

## 14. Conclusions

Personalized medicine represents an important evolution in pediatric urology, but its clinical value will depend less on the amount of information available than on the quality of the decisions that information enables. Individualized risk assessment should integrate clinical phenotype, imaging, functional measures, biomarkers, genomic information, and longitudinal observations when these data meaningfully improve prediction or management.

The current evidence is promising but remains immature. Many predictive and AI-based models require rigorous external validation, assessment of calibration and reproducibility, and, most importantly, demonstration of clinical utility and impact on patient outcomes before routine implementation. Multimodal approaches should therefore be evaluated not simply according to their technical performance, but according to whether they change clinical decisions in a way that benefits children.

Because pediatric urological conditions evolve with growth and development, longitudinal reassessment will remain an important component of personalized care. Ultimately, personalized pediatric urology should be understood as individualized risk assessment linked to clinically meaningful decisions: identifying which children require closer surveillance or intervention, which can safely avoid unnecessary investigations or treatment, and when management should be reconsidered. The goal is not more information for its own sake, but better decisions for the individual child.

## Figures and Tables

**Table 1:** From conventional assessment to personalized medicine in pediatric urology.

Domain	Established	Emerging	Future Direction
Risk assessment	Clinical, functional, anatomical, and longitudinal assessment	Multivariable risk models	Dynamic, continuously updated risk prediction
Phenotyping	Individualized clinical and functional phenotyping	Integration of biomarkers, imaging, and genomic data	Multimodal patient phenotyping
Imaging	Standard imaging interpreted in clinical context	Quantitative imaging and AI-assisted analysis	Automated multimodal imaging-based prediction
Biomarkers	Conventional laboratory assessment	Urinary and tissue biomarkers under investigation	Validated biomarker-guided decision-making
Genomics	Targeted genetic testing in selected patients	Broader genomic testing and genotype–phenotype correlation	Genomic risk stratification integrated into care
Follow-up	Risk-adapted surveillance and longitudinal reassessment	Prediction-supported follow-up	Dynamic model-informed surveillance
Surgery	Individualized surgical selection based on anatomy, function, symptoms, and expected benefit	Predictive models for complications and outcomes	Prospectively validated decision-support tools
AI	Limited routine clinical use	AI-based imaging and prediction models	Externally validated AI integrated into clinical workflows

AI—Artificial Intelligence.

**Table 2:** Examples of personalized decision-making across pediatric urology.

Condition	Traditional Decision Driver	Patient-Specific Factors	Personalized Clinical Question
Vesicoureteral reflux	Reflux grade	Febrile UTI, renal status, bladder and bowel dysfunction, age, laterality, clinical trajectory	Does this child need treatment, and if so, which treatment?
Hydronephrosis/UPJO	Degree of dilatation	Symptoms, drainage, differential renal function, parenchymal appearance, trajectory	Is the obstruction clinically significant, and when should intervention occur?
CAKUT	Anatomical diagnosis	Phenotype, bilateral involvement, renal function, extrarenal features, genotype	Is this an isolated anomaly or part of a broader disorder, and what is the long-term risk?
Hypospadias	Meatal position/anatomical severity	Penile size, curvature, tissue quality, glans configuration, previous surgery, functional goals	Which reconstruction best fits this child rather than this anatomical category?
Undescended testis	Testicular position	Age, position, size, laterality, associated anomalies, testicular development	What is the expected benefit of intervention for this individual child, and when?
DSD/complex genital anomalies	Anatomical phenotype	Genetic, endocrine, hormonal, reproductive, psychosocial and patient/family factors	What management strategy best addresses the child’s overall biological and functional phenotype?

UTI—urinary tract infection; CAKUT—congenital anomalies of the kidney and urinary tract; UPJO—ureteropelvic junction obstruction; DSD—disorders of sex development.

## Data Availability

No new data were created or analysed in this study. Data sharing is not applicable to this article.
